# Hypoxia-inducible factor prolyl hydroxylase 2 (PHD2) is a direct regulator of epidermal growth factor receptor (EGFR) signaling in breast cancer

**DOI:** 10.18632/oncotarget.14241

**Published:** 2016-12-27

**Authors:** Nina Kozlova, Marieke Wottawa, Dörthe Magdalena Katschinski, Glen Kristiansen, Thomas Kietzmann

**Affiliations:** ^1^ Faculty of Biochemistry and Molecular Medicine and Biocenter Oulu, University of Oulu, 90014 Oulu, Finland; ^2^ Institute of Cardiovascular Physiology, University Medical Center, Georg-August-University Göttingen, 37073 Göttingen, Germany; ^3^ Institute of Pathology, University Hospital Bonn, 53127 Bonn, Germany

**Keywords:** breast cancer, EGFR, PHD2, hypoxia signaling, EGFR-signaling

## Abstract

Clinical studies in breast cancer suggest important associations between intratumoral hypoxia, the upregulation of epidermal growth factor receptor (EGFR or HER1), hypoxia-inducible factor 1α (HIF-1α), and reduced patient survival. However, direct molecular links between EGFR and the hypoxia signaling system are not yet established. Since the oxygen sensor hypoxia-inducible factor prolyl hydroxylase 2 (PHD2) is considered to be the main HIF-1α regulator, we hypothesized that PHD2 and EGFR may be interconnected at the molecular level. By analyzing samples from 313 breast cancer patients, we found that EGFR is a first clinicopathological parameter positively correlating with PHD2. Mechanistically, we identified PHD2 as a direct binding partner of EGFR and show that PHD2 regulates EGFR stability as well as its subsequent signaling in breast carcinoma cells. Overall, we introduce for the first time the direct crosstalk between the oxygen sensor PHD2 and EGFR-mediated tumorigenesis in breast cancer.

## INTRODUCTION

The epidermal growth factor receptor EGFR (also known as ERBB or HER1) is a member of the ERBB cell-surface receptor tyrosine kinase family. EGFR is of immediate medical and biological importance due to its well-established roles in developmental biology, tissue homeostasis, and cancer [1]. Overexpression of EGFR was reported in 15-20% of all breast carcinomas and in 50-70% of triple negative breast cancers (TNBC) [2–5]. It is known that breast cancers with high EGFR expression are more aggressive, larger in size and more likely to metastasize to the lymph nodes [1] and brain [6]. Additionally, patients with EGFR-positive tumors have a worse overall, disease free and post-relapse survival after hormonal and/or chemotherapy [1].

The more rapid growth of EGFR-positive tumors is linked with intratumoral hypoxia and overexpression of the hypoxia-inducible transcription factor 1α (HIF-1α) [7]. The upregulated levels of HIF-1α in breast cancer are associated with high tumor grade, high proliferating microvessel density [8], increased rate of metastasis [9–12], as well as with a decreased breast cancer-specific survival [13]. Additionally, enhanced expression of HIF-1α has been shown to mediate resistance to chemotherapy and radiotherapy [14].

It was suggested that hypoxia induces expression of EGFR and its ligands [15, 16], and vice versa, EGFR signaling might enhance the cellular response to hypoxia by increasing expression of HIF-1α via the oncogenic PI3K/AKT and MAPK/ERK pathways [17–19]. Importantly, the levels of HIF-1α are regulated by prolyl-hydroxylases (PHDs), which hydroxylate proline residues within the HIF-1α subunit under normoxia, marking it for subsequent ubiquitination and proteasomal degradation [20]. Of the four PHDs known so far [21], PHD2 appears to be the main HIF-1α regulator and key oxygen sensor [22], which implies PHD2 may have a regulatory role in the pathogenesis of cancer. Indeed, recent studies suggested that PHD2 serves as potential tumor suppressor in breast cancer [13, 15]. Since the overexpression of EGFR and PHD2 in a hypoxic environment may have a profound role in breast tumor progression and metastases [4, 23–25], we hypothesized that there might be an association on a molecular level between PHD2 and EGFR. Therefore, we investigated how the expression of these two proteins is correlated in clinical samples of breast cancer patients and whether they may be directly linked. Our study describes for the first time a significant positive correlation between PHD2 and EGFR expression in 313 breast cancer patients. In addition, we identified PHD2 as a binding partner of EGFR and showed that PHD2 acts as a regulator of EGFR signaling and receptor stability in MDA-MB-231 breast carcinoma cells. Moreover, we introduce an additional level of crosstalk between hypoxia/PHD2-mediated signaling and EGFR-induced tumorigenesis in breast cancer, which is important for the development of novel breast cancer treatment options.

## RESULTS

### PHD2 levels positively correlate with EGFR levels in breast cancer

**Figure 1:**
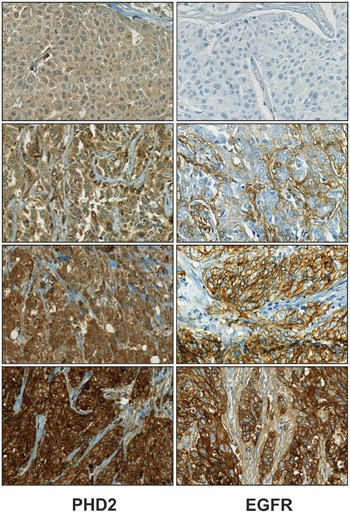
PHD2 and EGFR expression levels positively correlate in breast cancer Processed tissue microarrays of breast cancer biopsies from 313 patients were stained with PHD2 and EGFR antibodies (cf Materials and methods). Four representative immunohistochemistries of human breast cancer with low and high expression of PHD2 and EGFR are shown. Magnification 10x.

Since clinical studies support an important link between intratumoral hypoxia and upregulation of EGFR, we were interested to see whether there is a correlation between the major HIF-1α negative regulator PHD2 and EGFR levels. By analyzing PHD2 and EGFR expression in TMAs of tumor biopsies from 313 human breast cancer patients, we found that PHD2 showed a positive and significant correlation to EGFR expression (correlation coefficient = 0.231, p<0.001; n=313). In line with earlier studies, no significant correlations were found between PHD2 protein levels and HER2, ER or PR [15]. Representative immunological stainings illustrating the expression patterns of PHD2 (cytoplasmic) and EGFR (membranous) and the correlation of both proteins are shown in Figure 1.

### PHD2 directly interacts with EGFR

**Figure 2:**
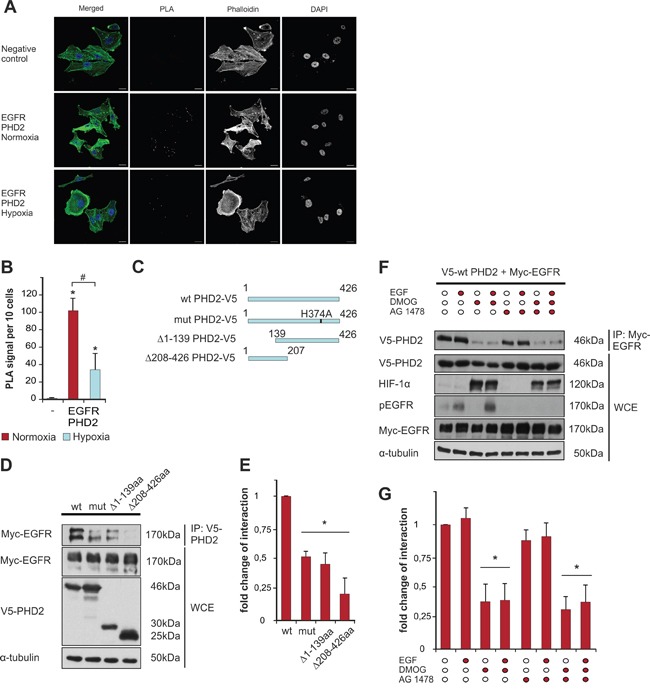
PHD2 interacts with EGFR **A**. Interaction of endogenous EGFR and PHD2 was visualized by proximity ligation assay (PLA) in MDA-MB-231 cells. Red spots reflect the interaction. MDA-MB-231 cells were cultured in full media and then exposed to either normoxic or hypoxic conditions, fixed and immunostained according to the Duolink manufacturer’s protocol (cf Materials and methods). Scale bars, 20 μm. **B**. Quantification of the number of PLA signals. * significant difference between the number of proximity ligation sites under normoxia/hypoxia vs. negative control, # significant difference between the number of proximity ligation sites under hypoxia vs. normoxia. **C**. Schematic presentation of the V5-tagged PHD2 proteins: wt PHD2, catalytically inactive PHD2 H374A (mut), PHD2 lacking amino acids 1-139 from the N-terminus (Δ1-139), and PHD2 variant lacking amino acids 208-426 from the C-terminus (Δ208-426). **D**. Western Blot analysis of immunoprecipitates (IP) and whole-cell lysates (WCE) from HEK-293 cells, overexpressing V5-tagged PHD2 variants (wt, mut, Δ1-139 or Δ208-426) and Myc-EGFR. Blots from anti-V5 IPs were probed with Myc-tag antibody. WCEs were probed with V5-tag, Myc-tag and α-tubulin antibodies. **E**. Quantification of the interaction between Myc-EGFR and V5-PHD2 variants. The levels of PHD2-bound Myc-EGFR from the lysates of HEK-293 cells, expressing wt PHD2 and Myc-EGFR (respective control) were set to 1. * significant difference between the Myc-EGFR levels from the lysates of HEK-293 cells, expressing mut, Δ1-139 or Δ208-426 PHD2 variants vs. control. **F**. Western Blot analysis of anti-Myc IPs and WCEs from HEK-293 cells, overexpressing wt V5-PHD2 together with Myc-EGFR and pre-treated with the PHD inhibitor DMOG (2 mM), the EGFR tyrosine kinase inhibitor AG 1478 (1 mM) or both followed by stimulation with either vehicle or EGF (100 ng/ml) for 10 min. Blots from IPs were probed with V5-tag antibody. WCEs were probed with V5-tag, HIF-1α, phospho-EGFR, Myc-tag and α-tubulin antibodies. **G**. Quantification of the interaction between Myc-EGFR and V5-PHD2 in the presence of EGF/DMOG/AG 1478. The levels of Myc-EGFR-bound V5-PHD2 from the lysates of non-treated HEK-293 cells (respective control) were set to 1. * significant difference treatment vs. control. Results are presented as mean values of three independent experiments ± SD. The statistical comparison between groups was performed by using Student’s two-tailed *t*-test. *p ≤ 0.05.

After seeing a positive correlation between PHD2 and EGFR in tumor biopsies, we sought to investigate whether PHD2 and EGFR undergo a direct interaction. To address this on the endogenous level we first used a proximity ligation immunoassay (PLA) in the MDA-MB-231 breast cancer cells. Since the availability of molecular oxygen is the predominant requirement for the activity of PHD2 [26], we performed PLA assays under both normoxic and hypoxic conditions. While we observed a strong dotted fluorescence signal indicating an interaction between endogenous EGFR and PHD2 under normoxic conditions, the number of proximity ligation sites under hypoxia was reduced by about 60% suggesting that PHD2 catalytic activity is important for the interaction (Figure 2A, 2B).

Next, we aimed to explore this novel finding in more detail. PHD2 is a protein of 426 amino acids with a hydroxylase subunit in the C-terminus (amino acids 291-392), while the N-terminus of the molecule is still rather poorly characterized. We used V5-tagged full length PHD2 and PHD2 mutants lacking catalytic activity PHD2 H374A, the N-terminus (PHD2 Δ1-139) or the C-terminus (PHD2 Δ208-426) (Figure 2C), along with Myc-tagged EGFR-expressing constructs for immunoprecipitation studies in HEK-293 cells. After verification of the V5-tag and Myc-tag antibody specificity for immunoprecipitation studies (Supplementary Figure 1A, 1B), we were able to show that full length PHD2 and the PHD2 variants interact with EGFR. In line with the results from the proximity ligation assay, the binding between EGFR and PHD2 H374A was reduced by about 50% compared to the binding between EGFR and wt PHD2. Additionally, when the PHD2 constructs lacking amino acids at the N-terminus, or the catalytic part, were employed in the assay, the binding between EGFR and these variants was reduced (Figure 2D-2G, Supplementary Figure 1C, 1D).

In many tumors EGF is produced either by the tumor cells themselves or is available from surrounding stromal cells, leading to constitutive EGFR activation [27]. Therefore, our next aim was to check if the binding between EGFR and PHD2 is not only dependent on PHD2 activity but also on the tyrosine kinase activity of EGFR. To address these, we performed immunoprecipitation experiments after stimulation of cells either with the vehicle or EGF for 10 min in the presence or absence of the EGFR tyrosine kinase inhibitor AG 1478 (Tyrphostin) or the prolyl hydroxylase inhibitor dimethyloxalylglycine (DMOG), or both simultaneously. Interestingly, the presence of EGF did not influence the interaction between PHD2 and EGFR (Figure 2F, 2G); however, there was a trend for decreased binding when cells were pre-treated with AG 1478. In line with the results of the PLA assay and immunoprecipitation studies using catalytically inactive PHD2 or PHD2 variants lacking the catalytic part (Figure 2D-2G, Supplementary Figure 1C, 1D), treatment with the hydroxylase inhibitor DMOG reduced the binding between EGFR and PHD2 by about 50% (Figure 2F, 2G). Notably, treatment of cells with DMOG and AG 1478 did not augment the reduction in binding between the two proteins. Collectively, these results indicate that the interaction between EGFR and PHD2 depends neither on the presence of EGF nor on the catalytic activity of EGFR itself, while the presence of the full-length catalytically active PHD2 is critical for the complex formation.

### Knockdown of PHD2 reduces EGFR levels

**Figure 3:**
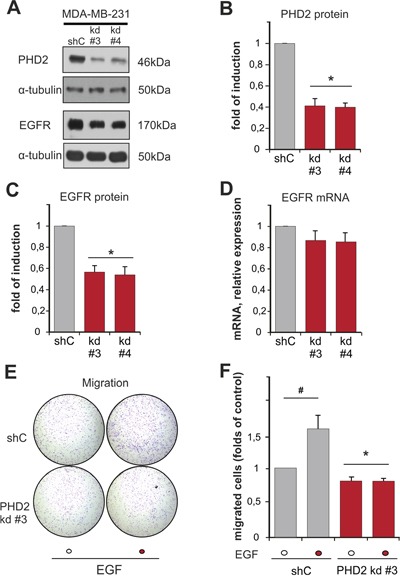
Knockdown of PHD2 in MDA-MB-231 cells affects EGFR levels and EGF-driven motility **A**. Representative immunoblots of PHD2 and EGFR levels in the lysates of MDA-MB-231 shC control and PHD2 knockdown clones #3 and #4. **B, C**. Quantification of PHD2 levels (B) and EGFR levels (C) in MDA-MB-231 cells. The PHD2 and EGFR levels in the shC (respective control) were set to 1. * significant difference between PHD2 and EGFR levels in the knockdown clones #3 and #4 vs. control. **D**. Quantification of EGFR mRNA levels in MDA-MB-231 cells. The EGFR mRNA levels in the shC (respective control) were set to 1.* significant difference between EGFR mRNA levels in the PHD2 knockdown clones #3 and #4 vs. control. **E**. MDA-MB-231 shC control and PHD2 knockdown cells #3 were treated with either vehicle or with EGF (100 ng/ml) for 2 days and cell migration was analyzed in a transwell migration assay. Representative photographs of the whole cell culture insert field from the migration assay. **F**. Quantification of the transwell migration assay. The number of migrated non-treated shC control cells was set to 1 (respective control). * significant difference between PHD2 knockdown cells vs. control. # significant difference EGF-treated cells vs. non-treated. In all experiments more than 500 cells were scored. Results are presented as mean values of three independent experiments ± SD. The statistical comparison between groups was performed by using Student’s two-tailed *t*-test. *p ≤ 0.05.

Our next interest was to find out whether these proteins have an influence on the expression of each other. To study this, we measured the expression of EGFR in two independent clones of MDA-MB-231 breast cancer cells with a stable knockdown of PHD2 (clones #3 and #4), which displayed a normoxic accumulation of HIF-1α and HIF-2 α as previously described (Supplementary Figure 2) [28]. Both, PHD2 knockdown clones #3 and #4, displayed a 50% lower content of EGFR than the scrambled (shC control) cells (Figure 3A–3C). Interestingly, PHD2 knockdown in these cells did not have an impact on EGFR mRNA levels (Figure 3D). Thus, these data indicate that lack of PHD2 affects EGFR levels on the post-transcriptional level. Since both of the PHD2 knockdown clones showed equally downregulated EGFR levels, we continued studying the impact of PHD2 on EGFR signaling in the PHD2 knockdown #3 cells.

To address the functional consequences of the lower EGFR levels in these PHD2 knockdown cells, we investigated EGF-dependent cell motility. Transwell migration assays showed that the basal motility of the PHD2 knockdown clone #3 was slightly but significantly lower than the motility of shC control cells (Figure 3E, 3F). Treatment of cells with EGF increased the number of migrated shC cells by about 1.5 fold compared to non-treated cells. By contrast, the PHD2 knockdown clone #3 was irresponsive to EGF treatment, which resulted in the same number of migrated cells regardless whether EGF treatment was present or not (Figure 3E, 3F). Together, these data show that PHD2 affects EGFR levels and, as a consequence, the response towards EGF.

### Knockdown of PHD2 affects EGFR activation in response to EGF

Since the above data indicated that PHD2 knockdown affects the cellular response to EGF we were next interested to see to what extent this has an impact on the EGFR signaling. First we checked the phosphorylation status of EGFR at Tyr1068, a site, critical for the activation of the MAPK pathway [27], in MDA-MB-231 shC and PHD2 knockdown cells treated with EGF for 1, 5, 10, 30 and 60 minutes.

**Figure 4:**
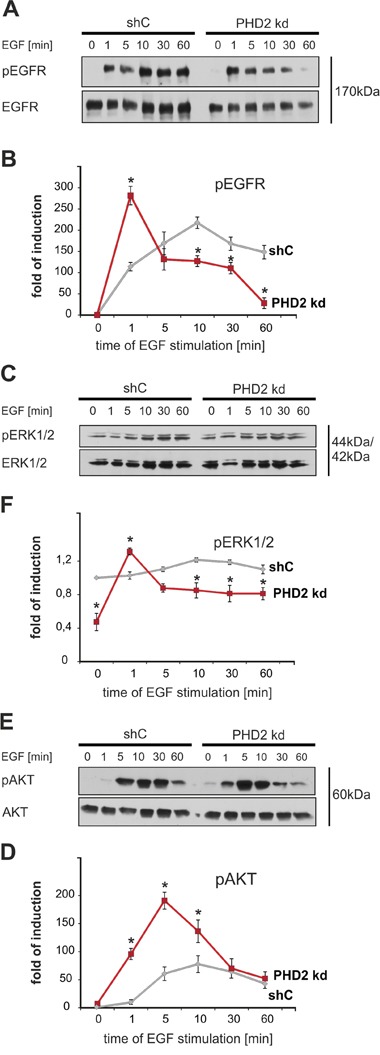
PHD2 knockdown leads to a less sustained activation of EGFR and its downstream signaling pathways MDA-MB-231 shC control and PHD2 knockdown cells were treated with either vehicle or EGF (100 ng/ml) for 0, 1, 5, 10, 30, 60 min. The phosphorylation of EGFR and kinases was measured with phosphospecific antibodies. **A**. Representative immunoblots of EGF receptor phosphorylation in MDA-MB-231 shC control and PHD2 knockdown cells. **B**. Quantification of EGFR activity. The ratio of phosphorylated EGFR to total EGFR levels in untreated shC control cells was set equal to 1. * significant difference PHD2 knockdown cells vs. same time point shC control cells. **C**. Representative immunoblots of ERK1/2 kinase activation in in MDA-MB-231 shC control and PHD2 knockdown cells. **D**. Quantification of ERK1/2 activity. The ratio of phosphorylated ERK1/2 to total ERK1/2 levels in untreated shC control cells was set equal to 1. * significant difference PHD2 knockdown cells vs. same time point shC control cells. **E**. Representative immunoblots of AKT kinase activation in MDA-MB-231 shC control and PHD2 knockdown cells. **F**. Quantification of AKT activity. The ratio of phosphorylated AKT to total AKT levels in untreated shC control cells was set equal to 1. * significant difference PHD2 knockdown cells vs. same time point shC control cells. Results are presented as mean values of three independent experiments ± SD. The statistical comparison between groups was performed by using Student’s two-tailed *t*-test. *p ≤ 0.05.

The phosphorylation of EGFR was already detectable at 1 minute after EGF stimulation in both the shC control and the PHD2 knockdown #3. However, the maximal EGFR activation in control cells became detectable 10 minutes after addition of EGF, while in PHD2 knockdown #3 cells EGFR activation reached its maximal peak already after 1 minute, was less pronounced compared to shC cells and declined thereafter. In control cells EGFR remained highly phosphorylated up to 60 minutes, whereas in PHD2 knockdown #3 cells EGFR activation was almost undetectable 60 minutes after stimulation (Figure 4A, 4B). Similarly PHD2 knockdown clone #4 reached its peak in EGFR activation at the same time point, declined also faster and was overall less apparent (Supplementary Figure 3A, 3B). These observations indicate that PHD2 knockdown leads to a less sustained activation of EGFR.

Next, we continued assaying major downstream EGFR signaling pathways MAPK/ERK and PI3K/AKT [29] by investigating the phosphorylation of ERK1/2 and AKT in MDA-MB-231 shC and PHD2 knockdown #3 cells treated with EGF as above. In PHD2 knockdown #3 cells activation of ERK1/2 was displayed 1 minute after EGF addition, thereafter ERK1/2 activation decreased. In shC control cells ERK1/2 reached its maximal activity within 10 minutes after EGF addition and remained active at a higher level than in PHD2 knockdown cells for 60 minutes (Figure 4C, 4D). Furthermore, PHD2 knockdown cells displayed a more rapid transient EGF-dependent activation of AKT when compared to shC control cells. AKT became activated in PHD2 knockdown cells within 1 minute after addition of EGF and reached its maximum after 5 minutes, then it declined. In shC control cells AKT activity became detectable after 5 minutes after addition of EGF, reached its peak within 10 minutes and remained almost at that level until 60 minutes (Figure 4E, 4F). Together, the maximal ERK1/2 and AKT activation in the PHD2 knockdown cells were gained in a shorter time compared to shC control cells (Figure 4). Collectively, these results demonstrate that PHD2 knockdown leads also to a less sustained activation of EGFR downstream signaling pathways.

### Knockdown of PHD2 fosters degradation of EGFR

**Figure 5:**
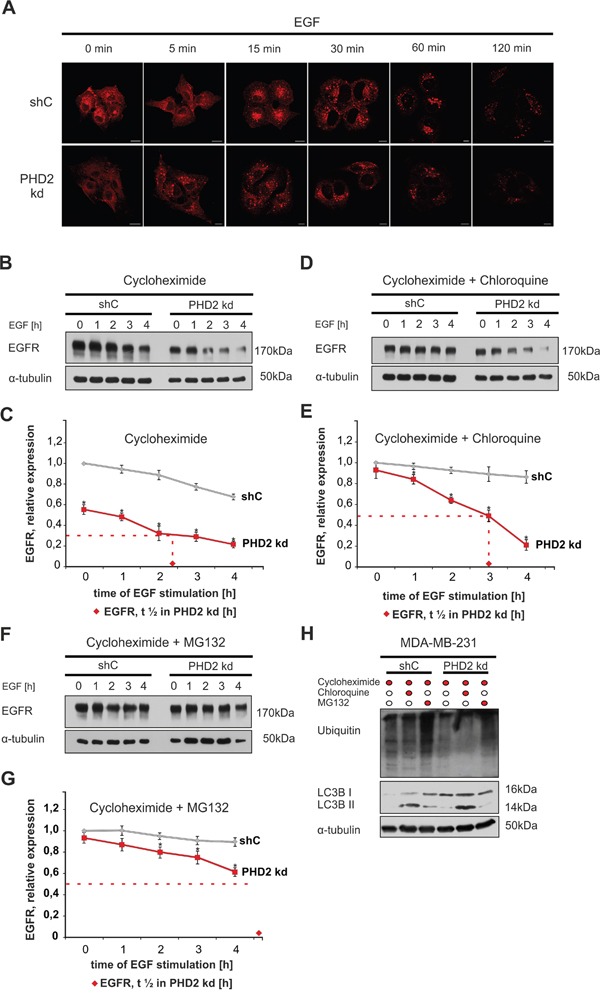
PHD2 knockdown fosters EGFR degradation **A**. Knockdown of PHD2 promotes EGFR internalization and endocytosis. MDA-MB-231 shC and PHD2 knockdown cells #3 were treated with EGF 100 ng/ml for 0, 5, 15, 30, 60 and 120 min. Fixed cells were immunostained with an EGFR antibody to follow EGF-induced receptor internalization and endocytosis. The EGFR signal was visualized using confocal microscopy (cf Materials and Methods). Scale bars, 20 μm. **B**. Representative immunoblots of EGFR levels in the lysates of MDA-MB-231 shC control and PHD2 knockdown cells, pre-treated with translation inhibitor cycloheximide (10 mM) for 12 h and treated with EGF (100 ng/ml) for 0, 1, 2, 3, 4 h. **C**. Quantification of EGFR levels in MDA-MB-231 cells treated with cycloheximide. **D**. Representative immunoblots of EGFR levels in the lysates of MDA-MB-231 shC control and PHD2 knockdown cells, pre-treated with cycloheximide (10 mM) and the lysosome inhibitor chloroquine (100 mM) for 12 h and treated with EGF (100 ng/ml) for 0, 1, 2, 3, 4 h. **E**. Quantification of EGFR levels in MDA-MB-231 cells treated with cycloheximide and chloroquine. **F**. Representative immunoblots of EGFR levels in the lysates of MDA-MB-231 shC control and PHD2 knockdown cells, pre-treated with cycloheximide (10 mM) for 12 h and with the proteasome inhibitor MG132 (10 mM) for 2h, then treated with EGF (100 ng/ml) for 0, 1, 2, 3, 4 h. **G**. Quantification of EGFR levels. The ratio of total EGFR to α-tubulin in non-treated shC control cells was set equal to 1. *significant difference PHD2 knockdown cells vs. shC control cells at the same time point. The statistical comparison between groups was performed by using Student’s two-tailed *t*-test. *p ≤ 0.05. The time needed to reach 50% of the EGFR starting level is referred to as half-life of the receptor, and is marked with ♦ in PHD2 knockdown #3 cells. **H**. Representative immunoblots of the ubiquitin and LC3B II levels in the lysates of cycloheximide, chloroquine and MG132 pre-treated shC control and PHD2 knockdown cells as verification of lysosomal and proteasomal inhibition.

Binding of EGF to EGFR leads to internalization of the receptor and trafficking via the endocytic pathway [30]. Since we have observed lower EGFR levels and altered dynamics of EGFR downstream signaling in PHD2 knockdown cells, we addressed whether PHD2 contributes to receptor turnover and stability. Therefore, we treated shC control and PHD2 knockdown cells for 0, 5, 15, 30, 60 and 120 minutes with EGF and visualized the differences in receptor internalization and endocytosis upon ligand binding by immunofluorescence. In line with the data from the western blot assays, the EGFR fluorescent signal was weaker in PHD2 knockdown cells. Both cell types showed a similar diffused cell surface receptor distribution in the absence of the ligand (Figure 5A). Already 5 minutes after addition of EGF, PHD2 knockdown cells showed a different receptor localization. While in the shC control cells EGFR was still evenly distributed all over the cell surface like the unliganded receptor, the PHD2 knockdown cells displayed formation of large endocytic vesicles in the perinuclear region. By contrast, it took 15 minutes after EGF treatment before endocytic vesicles became visible in the perinuclear region of shC control cells. The EGFR fluorescent signal was present up to 120 minutes after addition of EGF in the shC control cells, whereas in the PHD2 knockdown cells it was hardly detectable after 120 minutes, suggesting that the receptor gets endocytosed faster upon the partial absence of PHD2 (Figure 5A).

Binding of the ligand results in either degradation or recycling of EGFR [30]. Next, we decided to investigate if PHD2 contributes to ligand-induced EGFR degradation. To do this, we treated MDA-MB-231 shC and PHD2 knockdown cells with EGF for 0, 1, 2, 3, 4 h and then measured total EGFR levels in the obtained lysates. To eliminate the impact of *de-novo* EGFR synthesis both shC control cells and PHD2 knockdown #3 were pre-treated with the translational inhibitor cycloheximide before treatment with EGF. The time in which EGFR reached 50% of its starting level is referred to as half-life of the receptor. In the control shC cells liganded EGFR half-life was more than 4 h, while in PHD2 knockdown cells EGFR half-life was reduced to approximately 2.5 h (Figure 5B, 5C). Similarly, the second independent PHD2 knockdown clone #4 showed also a reduced EGFR half-life of about 2.5 h, compared to shC control cells (Supplementary Figure 3B).

Lysosome-mediated degradation is considered to be the major mechanism to downregulate ligand-activated EGFR [30]; however, proteasome-mediated degradation was reported to be important as well [31]. Therefore, we aimed to further investigate to which extent PHD2 knockdown affects lysosomal or proteasomal degradation of liganded EGFR. To do this, we pre-treated the cells simultaneously with either cycloheximide and chloroquine or cycloheximide and MG132, a proteasome inhibitor, before stimulation with EGF. Pre-treatment with cycloheximide and chloroquine almost compensated differences in basal EGFR levels between control and PHD2 knockdown cells, which suggests, that lower EGFR levels upon PHD2 knockdown can be a result of an upregulated lysosomal degradation of the receptor (Figure 5D, 5E). Simultaneous presence of cycloheximide and chloroquine almost completely blocked degradation of EGFR in the EGF-treated shC control cells, whereas it stabilized EGFR by increasing its half-life up to 3 h in the PHD2 knockdown cells. Pre-treatment of cells with cycloheximide and MG132 before stimulation with EGF again stabilized EGFR in the shC control cells. In the PHD2 knockdown cells inhibition of the proteasomal degradation by MG132 further prolonged the half-life of EGFR to ≥ 4 h (Figure 5F, 5G). The accumulation of LC3B II and ubiquitin after chloroquine and MG132 treatment in the lysates of shC control and PHD2 knockdown cells indicates an inhibited lysosomal and proteasomal function (Figure 5H). Together, these data suggest that the differences in the EGFR levels between shC control and PHD2 knockdown cells depend both on the lysosomal and proteasomal degradation of the receptor, whereas the faster EGFR turnover in the presence of EGF can be attributed to the proteasomal function.

## DISCUSSION

The current investigation describes for the first time a direct connection between the oxygen sensor PHD2 and EGFR as well as its subsequent signaling in breast cancer. We report a direct correlation between PHD2 and EGFR expression levels in tumor biopsies of 313 patients (Figure 1) and in MDA-MB-231 breast cancer cells. Mechanistically we identify PHD2 as a direct binding partner of EGFR; the binding required PHD2 but not EGFR activity (Figure 2). As a consequence of the binding, EGFR turnover, stability and signaling, as well as cancer cell motility were affected (Figure 3, 4, 5).

The importance of EGFR for the progression of a variety of epithelial cancers is well documented. However, its expression and signaling activity are required not only for the regulation of cellular proliferation, but also for the induction of metastasis and angiogenesis of the tumor [4, 23, 24]. The growth and angiogenesis of solid tumors are in turn tightly controlled by the availability of oxygen and components of the hypoxia signaling network, among which HIFs, PHDs and different HIF-inducible genes are the major players. The molecular crosstalk between hypoxia signaling and other major regulators of breast cancer pathogenesis, such as EGFR, is rather complex and multilayered. It has been reported, that HIF-1α and HIF-2α are able to enhance EGFR expression and activity [32] via an increase in the translational efficiency of EGFR mRNA [16] or via the attenuation of rabaptin-5 transcription, which leads to longer half-lives of activated EGFR due to delayed late endosomal EGFR sorting [33]. Therefore, we initially expected that the knockdown of PHD2 and, as shown previously [28], the stabilization of HIF-1α and HIF-2α in our cell model (Supplementary Figure 2) would favor the upregulation of EGFR. However, the experimental results were quite opposite. Along with the patient data we demonstrate, that the receptor levels are significantly lower in the MDA-MB-231 PHD2 knockdown cells compared to the shC control ones and that the observed change can be attributed to reduced EGFR protein stability. Therefore, it might be possible that the downregulation of EGFR levels upon PHD2 knockdown takes place without the influence of HIF-1α- or HIF-2α, but rather depends on the partial absence of the PHD2 protein itself. This might point to a HIF-independent action of PHD2, which may have an impact on the outcome in cancer. So far, only one study reported the participation of another PHD family member, PHD3, in EGFR stability in glioma [34]. Interestingly, in that study PHD2 was not reported to mediate any changes in EGFR signaling, which may suggest that the PHD2-dependent stabilization of EGFR signaling which we observe in our model might be breast tissue specific.

While several studies showed an association between HIF-1α overexpression and poor prognosis in breast cancer [8, 9, 11, 12, 35], a conclusive view on the role of PHD2 in cancer has not been reached. So far, PHD2-expression status and its influence on breast cancer disease was not found to correlate with either estrogen receptor (ER), progesterone receptor (PR), EGF receptor 2 HER2/neu (ERBB2) [13, 15] or p53, Ki67 and BCL2 [14]. A recent study on breast cancer showed significantly shorter survival times of patients with low-level PHD2 tumors [15], suggesting an oncoprotective role for this enzyme. In line, in gastric cancer PHD2 expression appeared to be a strong positive marker for patient survival [36]. By contrast, studies from a spontaneously arising PyMT-oncogene-driven breast cancer model showed that inhibition of PHD2 significantly decreased the number of metastases in the lung, supporting the pro-oncogenic role of PHD2 in breast cancer [25]. Studies on head and neck squamous cell carcinoma showed that high expression of PHD2 was associated with an aggressive phenotype [37] and high tumor PHD2 levels in hepatocellular carcinoma (HCC) were associated with higher tumor stage, larger tumor size, and worse overall patient survival [38]. Additionally, in non-small cell lung cancer (NSCLC) high levels of PHD2 were assessed as an independent negative prognostic factor for disease specific survival [39]. Moreover, a trend for decreased survival upon high PHD2 expression was reported in ampullary adenocarcinoma [40]. Since our present study describes PHD2 as an important regulator of sustained EGFR activity and stability, we rather favor the idea of PHD2 promoting EGFR-driven pathogenesis in breast cancer.

The latter view is supported by our previous studies from breast cancer cell lines showing that downregulation of PHD2 leads to an alteration of cellular proliferation and motility. For instance, when PHD2 knockdown clone #3 was subcutaneously injected into SCID mice, tumor growth was significantly delayed [28]. Lower levels of EGFR in these cells may be considered as an additional reason for the delayed tumor growth of the PHD2 knockdown cells in the xenograft assay (Figure 3A, 3B). This observation is further underlined in our present study, where we show that PHD2 knockdown led to an inhibition of EGF-driven migration (Figure 3E, 3F). Moreover, we have observed that PHD2 knockdown altered the dynamics of the activation of the two major EGFR signaling pathways: MAPK/ERK and PI3K/AKT [29], suggesting that PHD2 presence is necessary for the activation of the liganded EGFR and its downstream signaling pathways.

While observing a difference in a pattern of EGFR kinase activation upon PHD2 knockdown, we hypothesized that the binding between PHD2 and EGFR may be influenced by the presence of EGF. However, the direct binding between PHD2 and EGFR turned out to be neither dependent, nor influenced by the presence of EGF (Figure 2G, 2H). At the same time the inhibition of EGFR with one of the EGFR tyrosine kinase inhibitors AG 1478 did not decrease the binding between the two proteins, while treatment with DMOG reduced the binding drastically (Figure 2). In line, the proximity ligation assay indicated a reduced EGFR-PHD2 interaction under hypoxia (Figure 1) supporting the importance of the catalytically active PHD2 for the binding. Additionally, immunoprecipitation studies using different PHD2 variants lacking the N-terminal or C-terminal part further indicated that the binding between PHD2 and EGFR depends on the presence of the full length PHD2 (Figure 2). Together, these observations imply that the binding between PHD2 and EGFR is constitutive and requires the presence of full length catalytically active PHD2, rather than the presence of the EGFR ligand.

Since internalization and endocytic sorting are one of the principal mechanisms by which EGFR signaling is regulated [41, 42], we checked if PHD2 knockdown contributes to the speed of receptor turnover (Figure 5). Our data show, that PHD2 knockdown leads to faster EGFR internalization following ligand binding, faster formation of endocytic vesicles and enhanced receptor degradation. In turn, the shortened existence of internalized EGFR results in reduced length of EGFR signaling and EGF-induced cell migration. It is known, that the speed of EGFR internalization and sorting is regulated by multiple adaptor proteins like clathrin adaptor protein complex (AP2), growth factor receptor-bound protein 2 (GRB2), proto-oncogene tyrosine-protein kinase SRC, E3 ubiquitin ligase CBL, CBL-interacting protein of 85 kDa (CIN85), and multiple components of the endosomal sorting complex required for transport (ESCRT) [30]. We examined whether some of those components were downregulated in the PHD2-knockdown cells and we could not find any difference in the expression levels of SRC, CBL or CIN85 (Supplementary Figure 5).

Altogether, our study is the first one to describe the relations between PHD2 and EGFR in both preclinical and clinical models of breast cancer. We identify PHD2 as a novel contributor to EGFR signaling in breast cancer by describing its direct participation in the stability and activity of EGFR. Thus, our findings may help to consider use of PHD2 inhibitors together with anti-EGFR antibodies, which alone have limited therapeutical benefit for the treatment of triple negative breast cancers [4, 43].

## MATERIALS AND METHODS

### Tissue microarray analysis

Tissue micro arrays (TMAs) of biopsies from 313 invasive breast cancer cases diagnosed at the Institute of Surgical Pathology (University Hospital, Zurich, Switzerland), were studied as described [15]. Patient age at the time of diagnosis ranged from 26 to 98 years with a median of 61 years (mean 62). TMA sections were processed using an automated immunohistochemistry platform (Benchmark, Ventana, Roche) with the PHD2 antibody at 1:100 dilution (NB100-137, Novus Biologicals) [15] and the EGFR antibody (3C6, Ventana, Roche), prediluted [44].

### Chemicals

All biochemicals and enzymes were of analytical grade and were purchased from commercial suppliers: chloroquine, cycloheximide, dimethyloxalylglycine (DMOG), MG132 and recombinant human EGF were from Sigma-Aldrich; AG 1478 (Tyrphostin) was from Cell Signaling, BamHI and XbaI restriction enzymes were from Thermo Fisher Scientific.

### RNA extraction and qRT-PCR

Total RNA was isolated from cells with GenElute mammalian total RNA miniprep kit (Sigma-Aldrich). Reverse transcription (RT) was performed with 1 μg RNA using the first-strand cDNA synthesis kit (Quanta Bioscience, GE Healthcare). qRT-PCR was performed with cDNA diluted 1:25 and the iTaq Universal SYBR Green Supermix reaction kit (Biorad) in combination with the Applied Biosystems 7500 thermal cycler (Applied Biosystems). EGFR relative mRNA expression was determined using the ΔΔCt data analysis method [45] using the EGFR forward 5′-tgcgtctcttgccggaat-3′ and reverse 5′-ggctcaccctccagaaggtt-3′ primers [46]. The hypoxanthine phosphoribosyltransferase 1 (*HPRT1*) gene was used as housekeeping gene and its expression was assessed with the HPRT1 forward 5′–gtaattggtggagatgatctctcaact-3′ and reverse 5′-tgttttgccagtgtcaattatatcttc-3′ primers [47].

### Plasmids and site directed mutagenesis

pcDNA6A-Myc-EGFR was a gift from Mien-Chie Hung (Addgene plasmid #42665) [48]. The catalytically inactive PHD2 H374A variant was generated from the pcDNA3.1-PHD2-V5-6xHis backbone via site directed mutagenesis (QuickChange mutagenesis kit, Promega) and was described previously [49, 50]. PHD2 deletion mutants lacking amino acids 1-139 (PHD2 Δ1-139) and 208-426 (PHD2 Δ208-426) were generated with site directed mutagenesis of pcDNA3.1-PHD2-V5-6xHis creating additional BamHI and XbaI restriction sites in the coding sequence of *EGLN1* (PHD2), respectively. Primers were as follows: PHD2 Δ1-139 forward 5′-gggctcggcggtggatcccgacgccatgcccggcaaggagg-3′, PHD2 Δ1-139 reverse 5′-cctccttgccgggcatggcgtcgggatccaccgccgagccc-3′, PHD2 Δ208-426 forward 5′-gcacggcatctgtctagaggacgacttcctcggc-3′, PHD2 Δ208-426 reverse 5′-gccgaggaagtcgtcctctagacagatgccgtgc-3′. Afterwards, mutants were digested with BamHI (for PHD2 Δ1-139) and XbaI (for PHD2 Δ208-426), purified (Gel/PCR DNA fragments extraction kit, GeneAid), re-ligated, and transformed into XL-1 blue competent cells. Several clones were picked for plasmid propagation and restriction analysis. All constructs were verified by DNA sequencing.

### Cell culture

Human embryonic kidney 293 (HEK-293) and human breast carcinoma MDA-MB-231 cells were maintained in Dulbecco’s modified Eagle’s medium (DMEM) supplemented with 10% fetal bovine serum (FBS), 50 U/ml penicillin and 100 μg/ml streptomycin in a humidified atmosphere containing 16% O_2_, 5% CO_2_, 79% N_2_ at 37°C. The MDA-MB-231 shC control and PHD2 knockdown clones (#3 and #4) were generated via transduction with lentiviral particles expressing PHD2 shRNA and described previously [28]. When indicated, the cells were incubated under hypoxic conditions under 5% O_2_, 5% CO_2_ balanced with N_2_ for 6 h. All cell lines underwent mycoplasma testing before their use.

### Transwell migration assay

Cell migration assays were performed using 24-well cell culture inserts with 8 μm pores (Becton Dickinson) as described previously [51, 52]. Briefly, the MDA-MB-231 shC and PHD2 knockdown #3 cells were treated with vehicle or EGF (100 ng/ml) for 2 days. Then, 1×10^4^ cells were seeded on the upper wells of the 24-well chambers in the presence of 0.1% serum and EGF (100 ng/ml). The lower wells were filled with medium containing 5% FBS. After incubation for 12 h, cells that migrated out onto the lower surface of membranes were fixed in 4% paraformaldehyde (PFA), stained with 1% crystal violet and counted (more than 500 cells were scored in each experiment).

### Protein preparation, EGF treatment and western blotting

MDA-MB-231 cells (2×10^5^ cells per plate) were plated on 6 cm dishes and cultured for 2 days in full serum. Afterwards cells were lysed in lysis buffer [50 mM Tris–HCl, pH 7.5, 150 mM NaCl, 1% Triton X-100, 1 mM *o*-vanadate, 50 mM NaF, 2 mM EDTA, 1 mM PMSF, complete protease inhibitor cocktail tablet (Roche)], mechanically triturated through a 1 ml syringe, kept on ice for 10 min and centrifuged at 14 000 g for 20 min at 4°C [52, 53]. For the assessment of PHD2 knockdown on HIF-αs accumulation, MDA-MB-231 shC and PHD2 knockdown cells were incubated under hypoxic conditions as indicated above, levels of HIF-1α and HIF-2α were determined by Western blotting from total cell lysates. For time-course studies of EGFR, ERK1/2 and AKT activation, MDA-MB-231 shC and PHD2 knockdown #3 and #4 cells were cultured in starvation medium (DMEM containing 0.1% FCS) 24 h before the treatment. To assay the effect of EGF (100 ng/ml) on kinase activation, treatments were performed for 0, 1, 5, 10, 30 and 60 min. Total levels of EGFR, PHD2, phospho-EGFR, phospho-AKT and phospho-ERK1/2 were determined by Western blotting from total cell lysates. Proteins (20 μg per sample) were separated by electrophoresis on 7.5-10% polyacrylamide gels and transferred to nitrocellulose membranes. Membranes were incubated with dilutions of the following antibodies: phospho-AKT (pSer473) (#9271), AKT (#9272), phospho-EGFR (pTyr1068) (#2236), EGFR (#2232), phospho-ERK1/2 (pThr202/pTyr204) (#9101), ERK1/2 (#9107) (all Cell Signaling), HIF-1α (#610959, BD Biosciences), HIF-2α (#NB100-122, Novus Biologicals), PHD2 (#3293, Cell Signaling), α-tubulin (B-5-1-2) (#T5168, Sigma-Aldrich) primary antibodies overnight at 4°C. Levels of SRC, c-CBL and CIN85 expression in MDA-MB-231 cells were determined by Western blotting from total cell lysates. WB membranes were incubated with dilutions of the following antibodies: SRC (#2109S), c-CBL (#2747S), CIN85 (#12304S) (all Cell Signaling). Appropriate secondary antibodies (peroxidase-conjugated IgG (Biorad)) were used at 1:5000 dilutions. The ECL kit (GE Healthcare) was used for signal detection. Blots were quantified by densitometry with the Image Quant TL program (GE Healthcare); densitometry data were normalized to total protein levels or to α-tubulin.

### Immunoprecipitation

HEK-293 cells were transiently transfected with expression plasmids encoding Myc-tagged EGFR and V5-tagged PHD2 variants (wt PHD2, catalytically inactive PHD2 (mut H374A), PHD2 Δ1-139aa, and PHD2 Δ208-426aa) to investigate the binding preferences of PHD2 and EGFR. Immunoprecipitations were carried out as described [53]. Cells were harvested 48 h post-transfection, washed twice with ice-cold phosphate buffered saline (PBS) and lysed as described. For immunoprecipitation in the presence of EGF and/or catalytic inhibitors of EGFR and PHD2, cells were pre-treated with AG 1478 (1 mM) or DMOG (2 mM) for 6 h prior to vehicle/EGF (100 ng/ml) treatment for 10 min, then cells were lysed. Aliquots of cleared HEK-293 cell lysates containing 1 mg of total protein were mixed with protein G Sepharose beads (GE Healthcare) and Myc-tagged EGFR was immunoprecipitated with the Myc-Tag antibody (Cell Signaling #2278) or V5-tagged PHD2 proteins were immunoprecipitated with the V5 Tag antibody (Thermo Fisher, R960-25) at 4°C overnight. The next day the beads were washed 5 times with lysis buffer, immune complexes were then resolved on SDS-PAGE 7.5% or 12.5%, respectively and analyzed as below with antibodies against the Myc and V5 epitope. Lysates from EGF and/or inhibitor-treated cells were checked for phosphorylation of EGFR and accumulation of HIF-1α as a verification of the inhibition of PHD2 and EGFR.

### Proximity ligation assay

The EGFR-PHD2 interaction in MDA-MB-231 cells was detected with the Duolink PLA Kit (Olink Bioscience, Uppsala, Sweden: PLA probe anti-rabbit plus; PLA probe anti-mouse minus; Detection kit orange) according to the manufacturer’s protocol. Briefly, cells were plated on coverslips, grown, incubated under hypoxic or normoxic conditions for 6 h prior to fixation with 4% PFA and permeabilized with blocking buffer. The samples were incubated with the primary rabbit EGFR polyclonal antibody (1:200, #2232, Cell Signaling) and mouse monoclonal PHD2 antibody (1:20, sc-271835, Santa Cruz) diluted in blocking solution at room temperature (RT) for 1 h. In the negative control the PHD2 antibody was omitted. After the last washing step with buffer A, the samples were incubated with Alexa Fluor 488 conjugated phalloidin (A12379, Thermo Fisher Scientific) at RT for 20 min, washed with buffer B and then incubated with bisbenzimidine (1:5000, Hoechst stain, Sigma-Aldrich). Afterwards, the cells were mounted using Shandon Immumount mounting media (#9990402, Thermo Fisher Scientific). Confocal microscopy was performed using a Zeiss Observer Z1 equipped with a LSM 700 confocal unit, 63x PlanApo oil immersion objective and appropriate filter sets for Hoechst 405, Alexa Fluor 488, and Alexa Fluor 546, and Zen2009 software. Images were recorded in a Z-stack, further processed via ‘maximum intensity projection’ tool provided by Zen 2009 software. In order to acquire a single channel image, channels were splitted using ImageJ software.

### Fluorescence microscopy

To visualize the downregulation of active EGFR, MDA-MB-231 shC cells and PHD2 knockdown #3 cells were plated on glass coverslips, starved for 16 h and treated with EGF (100 ng/ml) for 0, 5, 15, 30, 60 and 120 min. Cells were rinsed with PBS, fixed with 4% PFA, kept in blocking buffer (1xPBS, 1% BSA, 0.1% Saponin) for 60 min and further incubated with rabbit EGFR primary antibody (1:250, #2232, Cell Signaling) for 60 min. Coverslips were washed with blocking buffer and incubated with goat anti-rabbit, Alexa Fluor 546 conjugated secondary antibodies (1:500, A-11035, Thermo Fisher Scientific) at RT for 60 min. Coverslips were washed 3 times in PBS, once with water, and mounted using Shandon Immumount mounting media. The EGFR signal was visualized using by confocal microscopy as described above.

### EGFR half-life studies

MDA-MB-231 shC and PHD2 knockdown #3 cells (2×10^5^ cells per plate) were plated on 6 cm dishes and cultured for 1 day in full serum. 16 h before treatment, the cells were cultured in starvation medium (DMEM containing 0.1% FBS), supplemented with either cycloheximide (10 mM), or cycloheximide (10 mM) together with the lysosome inhibitor chloroquine (100 mM) for 16 h. To inhibit the proteasome, the cells were starved and pre-treated with cycloheximide for 16 h, followed by 2 h pre-treatment with MG132 (10 mM). Afterwards the cells were treated with EGF (100 ng/ml) for 0, 1, 2, 3 and 4 h, cell lysates were prepared as described above. To verify lysosomal and proteasomal inhibition, lysates from treated cells were checked for accumulation of LC3B II (#2775, Cell Signaling) and ubiquitin (sc-9133, Santa Cruz).

### Statistical analysis

The results are presented as means ± SD of at least 3 independent experiments. The statistical analyses were performed using Student's two-tailed *t*-test. Differences of p ≤ 0.05 were considered statistically significant. For statistical analysis of TMA-based expression data, spearman rank correlations were calculated [28].

## SUPPLEMENTARY FIGURES


