# Supplementary material for: Hypoxia-inducible factor prolyl hydroxylase 2 (PHD2) is a direct regulator of epidermal growth factor receptor (EGFR) signaling in breast cancer
Source: Oncotarget. 2016 Dec 27;8(6):9885–98. doi: 10.18632/oncotarget.14241 (PMC5354778; doi:10.18632/oncotarget.14241)
Supplement: Supplementary file 1 [file oncotarget-08-9885-s001.pdf]

Hypoxia-inducible factor prolyl hydroxylase 2 (PHD2) is a direct regulator of epidermal growth factor receptor (EGFR) signaling in breast cancer

SUPPLEMENTARY FIGURES

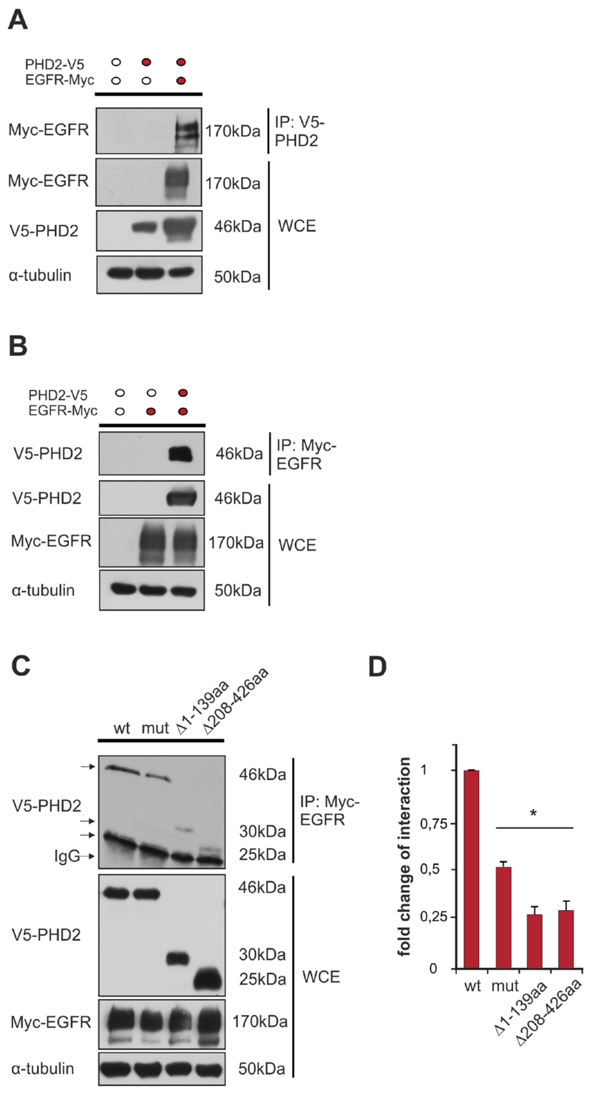

Supplementary Figure 1: Interaction of Myc-EGFR and V5-PHD2 in HEK-293 cells. The specificity of V5-tag and Myc-tag antibodies used for the detection of EGFR-PHD2 interaction was verified by Western Blot analysis of immunoprecipitates (IP) and whole-cell extracts (WCE) from non-transfected HEK-293 cells, and HEK-293 cells overexpressing either V5-tagged PHD2 and/or Myc-EGFR. **A.** Blots from anti-V5 IPs were probed with Myc-tag antibody. WCEs were probed with V5-tag, Myc-tag and α-tubulin antibodies. **B.** Blots from 'reverse' anti-Myc IPs were probed with V5-tag antibody. WCEs were probed with V5-tag, Myc-tag and α-tubulin antibodies. **C.** WB analysis of immunoprecipitates (IP) and whole-cell extracts (WCE) from HEK-293 cells, overexpressing V5-tagged PHD2 variants and Myc-EGFR. Blots from anti-Myc IPs were probed with V5-tag antibody. WCEs were probed with V5-tag, Myc-tag and α-tubulin antibodies. **D.** Quantification of the interaction between Myc-EGFR and V5-PHD2 variants from the 'reverse' immunoprecipitation. The levels of Myc-EGFR-bound wt V5-PHD2 from the lysates expressing wt PHD2 and Myc-EGFR (respective control) were set to 1. * significant difference between the precipitated V5-PHD2 levels from the lysates of HEK-293, expressing mut, Δ1-139 or Δ208-426 variants vs. control.

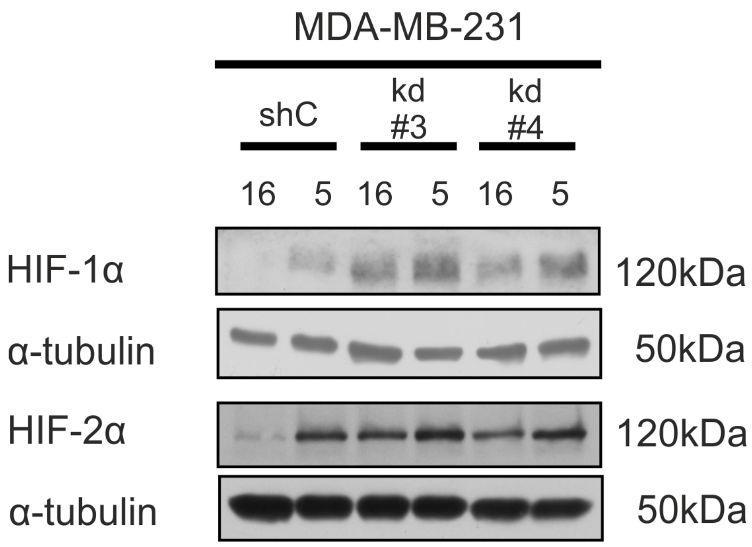

Supplementary Figure 2: MDA-MB-231 PHD2 knockdown clones #3 and #4 exhibit normoxic accumulation of HIF-1α and HIF-2α levels. WB analysis in the WCEs of MDA-MB-231 shC, #3, and #4 cells cultured under normoxia (16% O_2) or hypoxia (5% O_2) for 6 h. WCEs were probed with HIF-1α, HIF-2α, and α-tubulin antibodies.

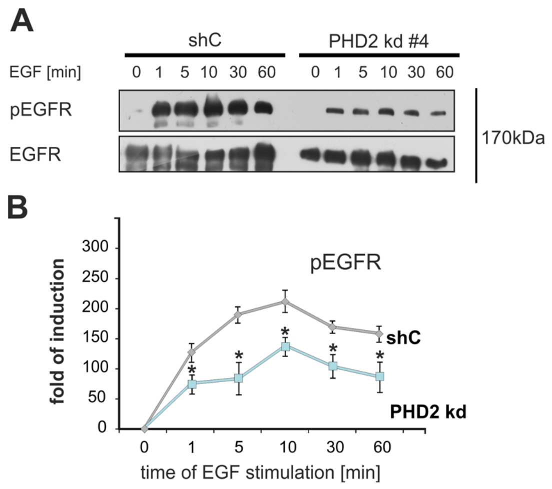

Supplementary Figure 3: PHD2 knockdown leads to a less sustained activation of EGFR in PHD2 knockdown clone #4. MDA-MB-231 shC control and PHD2 knockdown #4 cells were treated with either vehicle or EGF (100 ng/ml) for 0, 1, 5, 10, 30, 60 min. The phosphorylation of EGFR was measured with a phosphospecific antibody. **A.** Representative immunoblot of EGF receptor phosphorylation in MDA-MB-231 shC control and PHD2 knockdown cells. **B.** Quantification of EGFR activity. The ratio of phosphorylated EGFR to total EGFR levels in untreated shC control cells was set equal to 1. * significant difference PHD2 knockdown cells vs. same time point shC control cells.

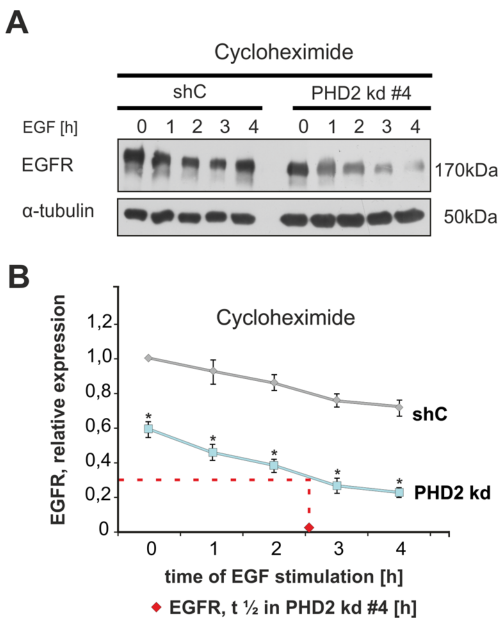

Supplementary Figure 4: PHD2 knockdown fosters EGFR degradation in PHD2 knockdown clone #4. A. Representative immunoblots of EGFR levels in the lysates of MDA-MB-231 shC control and PHD2 knockdown #4 cells, pre-treated with translation inhibitor cycloheximide (10 mM) for 12 h and treated with EGF (100 ng/ml) for 0, 1, 2, 3, 4 h. **B.** Quantification of EGFR levels in MDA-MB-231 cells treated with cycloheximide. The ratio of total EGFR to α-tubulin in non-treated shC control cells was set equal to 1. *significant difference PHD2 knockdown cells vs. shC control cells at the same time point. The statistical comparison between groups was performed by using Student's two-tailed *t*-test. *$p \leq 0.05$. The time needed to reach 50% of the EGFR starting level is referred to as half-life of the receptor, and is marked with ♦ in PHD2 knockdown #4 cells.

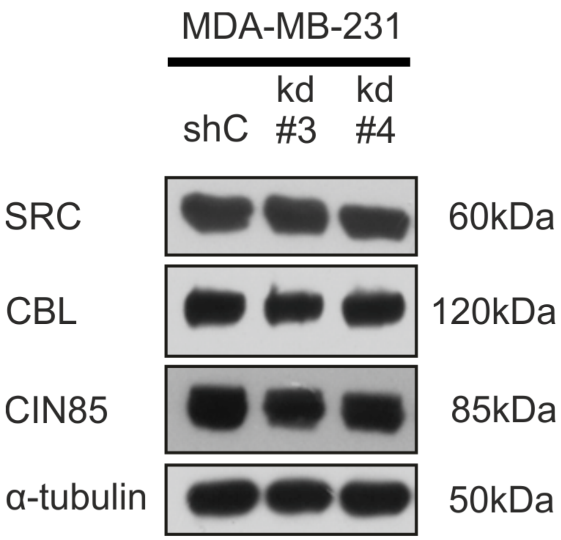

Supplementary Figure 5: PHD2 knockdown does not influence the expression levels of SRC, CBL and CIN85. Representative immunoblots of the S, CBL, CIN85 in the lysates of MDA-MB-231 shC control and PHD2 knockdown #3 and #4 cells.
